# Curcumin inhibits cholesterol uptake in Caco-2 cells by down-regulation of NPC1L1 expression

**DOI:** 10.1186/1476-511X-9-40

**Published:** 2010-04-19

**Authors:** Dan Feng, Lena Ohlsson, Rui-Dong Duan

**Affiliations:** 1 Gastroenterology and Nutrition Laboratory, Biomedical Center B11, Institution of Clinical Sciences, University of Lund, Lund, Sweden

## Abstract

**Background:**

Curcumin is a polyphenol and the one of the principle curcuminoids of the spice turmeric. Its antioxidant, anti-cancer and anti-inflammatory effects have been intensively studied. Previous in vivo studies showed that administration of curcumin also decreased cholesterol levels in the blood, and the effects were considered to be related to upregulation of LDL receptor. However, since plasma cholesterol levels are also influenced by the uptake of cholesterol in the gut, which is mediated by a specific transporter Niemann-Pick Cl-like 1 (NPC1L1) protein, the present study is to investigate whether curcumin affects cholesterol uptake in the intestinal Caco-2 cells.

**Methods:**

Caco-2 cells were cultured to confluence. The micelles composed of bile salt, monoolein, and ^14^C-cholesterol were prepared. We first incubated the cells with the micelles in the presence and absence of ezetimibe, the specific inhibitor of NPC1L1, to see whether the uptake of the cholesterol in the cells was mediated by NPC1L1. We then pretreated the cells with curcumin at different concentrations for 24 h followed by examination of the changes of cholesterol uptake in these curcumin-treated cells. Finally we determined whether curcumin affects the expression of NPC1L1 by both Western blot analysis and qPCR quantification.

**Results:**

We found that the uptake of radioactive cholesterol in Caco-2 cells was inhibited by ezetimibe in a dose-dependent manner. The results indicate that the uptake of cholesterol in this study was mediated by NPC1L1. We then pretreated the cells with 25-100 μM curcumin for 24 h and found that such a treatment dose-dependently inhibited cholesterol uptake with 40% inhibition obtained by 100 μM curcumin. In addition, we found that the curcumin-induced inhibition of cholesterol uptake was associated with significant decrease in the levels of NPC1L1 protein and NPC1L1 mRNA, as analyzed by Western blot and qPCR, respectively.

**Conclusion:**

Curcumin inhibits cholesterol uptake through suppression of NPC1L1 expression in the intestinal cells.

## Introduction

Elevated plasma cholesterol levels constitute a major risk factor for atherosclerosis and coronary heart diseases [1]. The levels of plasma cholesterol are influenced by de novo biosynthesis, absorption in the gut, and the removal of cholesterol from the blood [2]. The intestine plays a major role in regulating cholesterol homeostasis and about 36% reductions of plasma cholesterol could be achieved by total inhibition of cholesterol absorption [3]. Absorption of cholesterol is a multi-step process in which cholesterol is micellized by bile acids in the intestinal lumen, taken up by the enterocytes, assembled into lipoproteins, and transported to the lymph and the circulation. Niemann-Pick C1-like 1(NPC1L1) protein has been identified as a specific transporter for cholesterol uptake at the surface of plasma membrane [4]. Ezetimibe is a well-known inhibitor of NPC1L1 and has been widely used as an effective cholesterol-lowering drug for treating patients with hypercholesterolemia[5].

Curcumin is the major constituent of turmeric curcuminoids and has been found to have antioxidant, anti-tumor, anti-inflammatory properties [6-8]. Besides these well-known effects, curcumin was also found to affect lipid metabolism. More than 30 years ago, Rao et al showed that administration of curcumin decreased cholesterol levels in the blood and liver in normal animals [9]. Similar reductions were also identified thereafter in diabetic animals and animals fed high fat [10-12] and in healthy humans, varied with the dose, age and the period of administration [13-15]. The mechanism underlying the hypocholesterolemic effect may be related to the upregulation of LDL receptor [16,17]. Since plasma cholesterol levels are also influenced by absorption of cholesterol in the gut, we, in the present study, addressed a question whether curcumin affects the cholesterol uptake in the enterocytes.

## Materials and methods

### Materials

Caco-2 cells were obtained from American Tissue Culture Collection. The Modified Eagle Medium (DMEM), M199 medium, heat-inactivated fetal bovine serum (FBS), 1% non-essential amino acids were purchased from either Invitrogen or Sigma-Aldrich (Stockholm, Sweden). [^14^C] cholesterol (50 mCi/mmol) was purchased from American Radiolabeled Chemicals Inc (St. Louis, MO, USA). Curcumin (purity > 98%) was purchased from Sigma-Aldrich. NPC1L1 antibody was purchased from Santa Cruz (Santa Cruz, USA). QT-PCR kit was obtained from Bio-Rad (Stockholm, Sweden). Primers used in quantification of mRNA of NPC1L1 by qPCR were synthesized from DNA Technology (Rysskov, Denmark). Ezetimibe (purity >95%) was kindly provided by Schering-Plough Research Institute (Kenilworth, USA).

### Preparation of delipidized fetal bovine serum and cholesterol micellar solutions

The preparation of delipidized FBS was according to Gibson *et al *[18]. In brief, 20 g thixotropic gel powder (Cab-o-sil, Kodak) was added to 1 liter FBS and stirred overnight at 4°C. The mixture was then centrifuged at 15,000 rpm at 4°C for 1 h and the supernatant was collected and sequentially filtered through 0.20 μm filter. For preparing micellar cholesterol solutions, M199 culture media containing 3 mM sodium taurocholate, 30 μM monoolein and 1 nM [^14^C]cholesterol (1.25 × 1 0^5 ^dpm) were mixed and sonicated, as reported by Field et al [19]. The micellar solution was then passed through a 0.20 μm filter and kept at 37°C until use.

### Cell culture and stimulation

Caco-2 cells were cultured in 6 well plate in DMEM, containing 10% FBS, 1% penicillin-streptomycin, 2 mM L-glutamate, 1% non-essential-amino acids to confluence as described by Eckhardt et al [20]. Prior to experiment, the culture medium with 10% FBS was replaced with the medium containing the delipidized FBS, followed by incubating the cells for 24 h. The cells were then washed three times with M199 buffer, and then incubated with fresh medium containing the cholesterol micelles for 2 h. The medium was then removed and the cells were washed three times with ice-cold PBS. After centrifugation, the cell pellets were dissolved in 0.5 ml of 0.1 M NaOH and an aliquot of 0.1 ml of the lysate was taken for liquid scintillation counting. The protein concentration of the lysate was quantified by a kit from Bio-Rad and the results of incorporated cholesterol into Caco-2 cells were expressed as dpm/mg protein. To confirm that the uptake of cholesterol under this experimental conditions was mediated by NPC1L1 not passive diffusion, some cells were pretreated with ezetimibe, the inhibitor of NPC1L1, for 2 h. The changes of cholesterol uptake induced by ezetimibe were examined.

To study the effects of curcumin on cholesterol uptake, curcumin was freshly dissolved in dimethyl sulfoxide (DMSO) at different concentrations and immediately diluted in the DMEM culture medium as described above. The cells were pretreated with curcumin with different concentrations for 24 h. The final concentration of DMSO in the medium was 0.1% and the control cells were incubated with 0.1% DMSO only. After the pretreatment, the medium was removed and the cells were washed three times with ice-cold PBS, followed by incubation with the cholesterol micelles as described above. The cholesterol uptakes in the cells with and without curcumin treatment were compared.

### RNA estimation by real time quantitative RT-PCR(QT-PCR)

RNA was extracted from the cells using TRIzol reagents and converted to cDNA by a kit from Fermentas (Helsingborg, Sweden). The primers used in qPCR to quantify the mRNA of NPC1L1 were shown in Table 1. The cDNA isolated from the cells was mixed with the appropriate primers and 2 × SYBR Green PCR master mix (Bio-Rad) in 20 μl. Real-time RT-PCR was performed using the Bio-Rad iCycler system. The thermal cycler program was as follows: holding for 3 min at 95°C for one cycle, followed by amplification of cDNA for 45 cycles with melting for 15 s at 95°C, and annealing and extension for 1 min at 60°C. The mass of PCR products generated was estimated after each PCR cycle, and threshold cycle number is determined in the exponential phase of the curve. The values were normalized using GAPDH as an endogenous internal standard. The relative expression of the gene was calculated using the comparative threshold cycle (Ct) method.

**Table 1:** Primers used in this study.

Primer	Sequence
Human NPC1L1-F	5'-TATGGTCGCCCGAAGCA-3'
Human NPC1L1-R	5'-TGCGGTTGTTCTGGAAATACTG-3'
GAPDH-F	5'-CATGAGAAGTATGACAACAGCCT-3'
GAPDH-R	5'-AGTCCTTCCACGATACCAAAGT-3'

### Western blot

The cells were lysed and the cell free extract was prepared as described [21]. Proteins (40 μg) in cell lysate were subjected to 7.5% SDS PAGE. The resolved proteins in the gel were transferred to a nitrocellulose membrane electrophoretically overnight. The membranes were incubated with anti-NPC1L1 antibody (1:5000) and then with second antibody (1:50000) conjugated with horseradish peroxidase. The specific NPC1L1 bands (145 kD) were identified by enhanced chemiluminescence advance reagent. The membranes were then stripped and re-probed with anti-actin antibody as a loading control.

### Statistical analysis

The results are presented as mean ± S.E.M. Statistical analyses were performed using one-way analysis of variance (ANOVA) followed by the Bonferroni posttest for multiple comparisons. Differences were considered significant at *P *< 0.05.

## Results

### Uptake of cholesterol in Caco-2 cells was inhibited by ezetimibe

**Figure 1:**
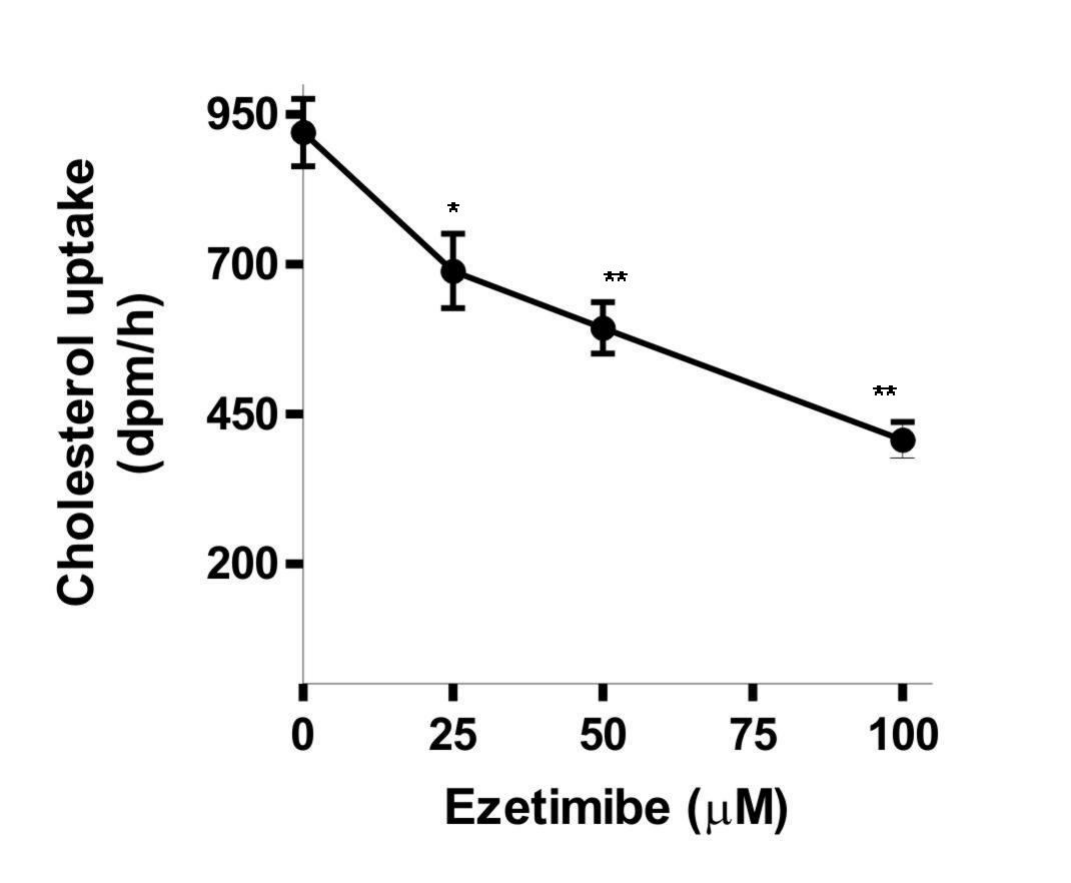
**Cholesterol uptake by Caco-2 cells is mediated by NPC1L1**. The cells were cultured to 100% confluence and pretreated with ezetimibe at different concentrations for 2 h. The cells were then incubated with radioactive micellar cholesterol for 2 h. The cells were then lysed and the uptake of radioactive cholesterol was quantified by liquid scintillation. Results are mean ± SEM of duplicate determinations in three separate experiments. * *P *< 0.05, ** *P *< 0.01.

Because uptake of cholesterol in the intestinal tract was mediated by NPC1L1 whose functions can be inhibited specifically by ezetimibe [4,5], we first examined whether cholesterol uptake in Caco-2 cells under the experimental conditions was related to the functions of NPC1L1. The answer is positive. As shown in Fig. 1, the cholesterol uptake was dose dependently inhibited by ezetimibe.

### Curcumin inhibited cholesterol uptake in Caco-2 cells

**Figure 2:**
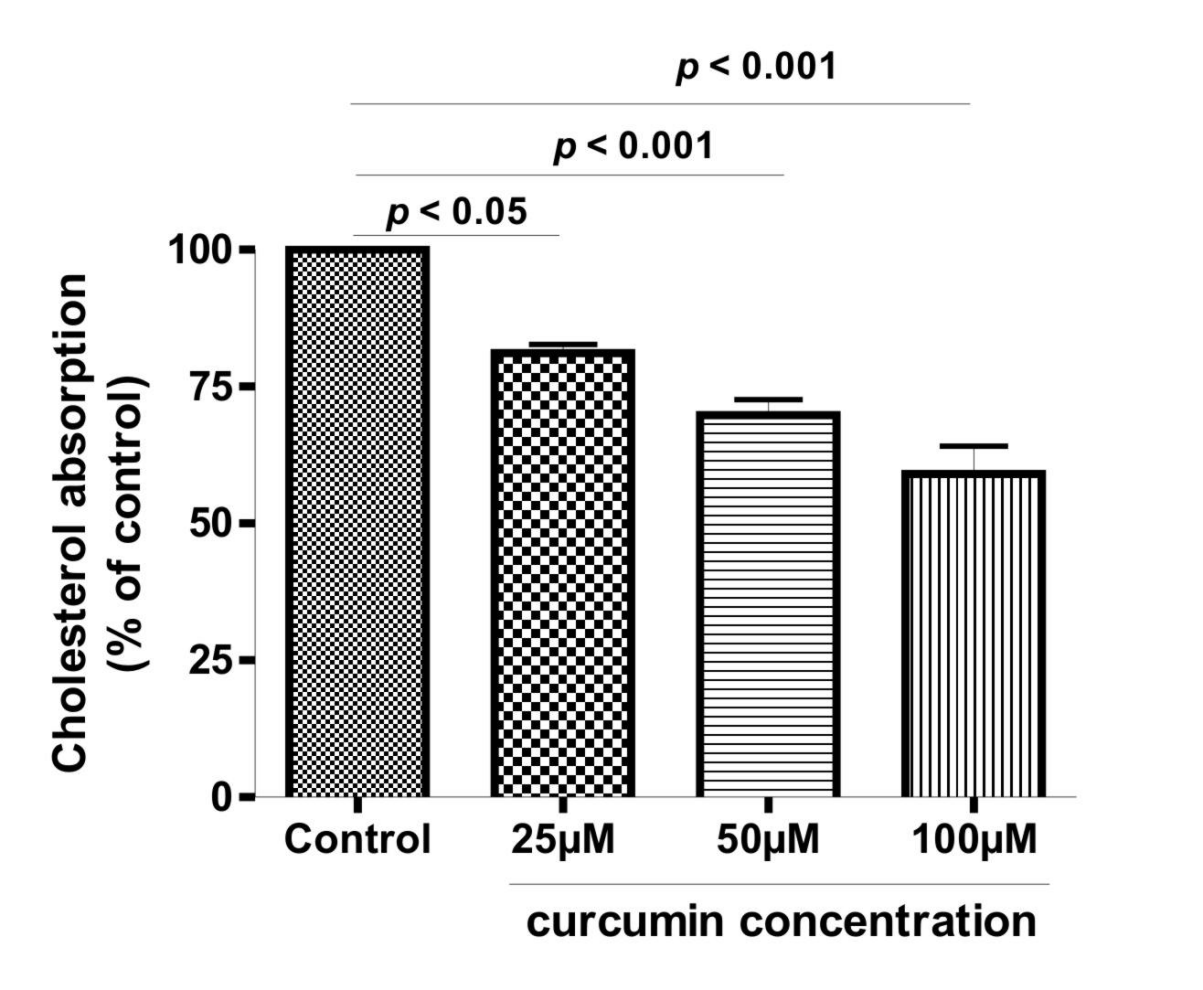
**Inhibitory effect of curcumin on micellar cholesterol absorption in Caco-2 cells**. The cells were pretreated with curcumin at different concentrations for 24 h, then incubated with radioactive micellar cholesterol for 2 h. The uptake of cholesterol in the absence of curcumin was normalized to 100%. Results are mean ± SEM from triplicate determinations in three separate experiments.

We then addressed a question whether curcumin can inhibit cholesterol uptake. After pretreating the cells with curcumin for 24 h, we found that cholesterol uptake in Caco-2 cells was decreased with the increasing concentrations of curcumin (Fig. 2). The threshold concentration of curcumin is about 25 μM and at 100 μM curcumin decreased cholesterol uptake by about 40%.

### The effects of curcumin were associated with decreased NPC1L1 protein expression

**Figure 3:**
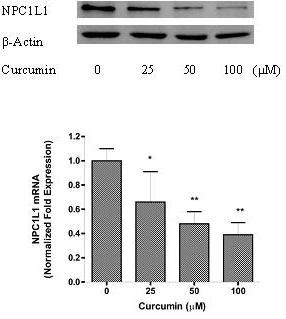
**The effect of curcumin on NPC1L1 protein expression in Caco-2 cells**. In the upper panel, the cells were treated with curcumin at different concentrations for 24 h, and the whole-cell lysates were analyzed by Western blot. The results are representative of three independent experiments. In the lower panel, NPC1L1 mRNA abundance was determined by real-time RT-PCR as described in Methods. Expression values were normalized to housekeeping genes, and expression in untreated cells was set to 1. Values shown represent means ± SEM of three independent experiments, * *P *< 0.01, ** *P *< 0.001 compared to untreated cells.

We then further studied whether curcumin could influence the levels of NPC1L1 in the intestinal cells. As shown in upper panel of Fig. 3, pretreatment of the cells with different concentrations of curcumin for 24 h significantly attenuated NPC1L1 protein levels. No similar changes could be identified for the levels of actin. To distinguish whether the effect was caused by an increased degradation or a decreased biosynthesis, the levels of mRNA of NPC1L1 after treating the cells with curcumin were quantified. As shown in the lower panel of Fig. 3, curcumin induced a dose-dependent reduction of NPC1L1 mRNA, as normalized with that of control gene GAPDH. About 50% inhibition could be identified by 50 μM curcumin.

## Discussion

Previous studies in animals and humans have shown that administration of curcumin decreased the cholesterol levels in blood (11-14). The mechanism underlying the effects was considered to be related to the increased expression of LDL receptor [16,17]. Since intestinal absorption of cholesterol also affects the levels of cholesterol in the blood, whether uptake of cholesterol in the enterocytes can be affected by curcumin is therefore an important question to be studied. Our results for the first time show that curcumin has inhibitory effects on cholesterol uptake and the inhibition is mediated by down-regulation of NPC1L1 expression. The conclusion is supported by the evidence from 3 lines in the present study. First, the uptake of cholesterol into Caco-2 cells was a process mediated by NPC1L1, since it could be inhibited in a dose-dependent manner by ezetimibe, a specific inhibitor of NPC1L1. Second, the NPC1L1 protein in the cells was decreased by treating the cells with curcumin. And third, the reduced NPC1L1 protein was most likely resulted from a decreased expression, as a significant decrease of NPC1L1 mRNA was demonstrated by curcumin treatment in a dose dependent manner.

Curcumin is one of the most intensively studied plant components and most of the studies are focused on its antiinflammatory and anticancer effects [22-24]. However, its hypocholesterolemic effects have documented for more than 30 years [9-15]. Most of the previous studies were performed in animal models followed by analysis of the cholesterol levels in the blood and liver. The most consistent finding from the previous studies is a decreased cholesterol level in the blood. The mechanism for such a decrease was suggested to be mediated by an upregulation of LDL receptor [16,17]. The present work demonstrated a novel effect of the component in the gut, i.e. to inhibit the expression of NPC1L1 in the enterocytes. Our results indicate that curcumin can decrease the cholesterol levels by two mechanisms, one in the intestine to inhibit the uptake via NPC1L1 transporter, and the other in the blood to increase the clearance by LDL receptor. Considering the poor absorption in the gut and low concentration of curcumin in the blood [24], its effect in the intestinal tract might be of more physiological implications than its systemic effects.

NPC1L1 is a crucial transporter for cholesterol uptake. Understanding the regulation of its expression is of importance in human health and disease. It is only recently that several studies showed that diet with low cholesterol and low fat increased the NPC1L1 expression in the small intestine in hamster [25]. A few dietary factors such as phytosterol [26], PUFA [27] and dietary supplement probiotics [28] may also affect expression of NPC1L1. Although the molecular signals regulating the expression of NPC1L1 is not clear, several nuclear receptors such as sterol responsive element binding protein 2(SREBP2), LXR and HNF4α might be involved [29]. Overexpression of SREBP2 results in an enhanced transcription of NPC1L1. Curcumin has been shown to affect the functions of about 50 transcriptional factors [22,30]. Whether some of these factors are linked to the nuclear receptor, leading to the inhibition of the expression of NPC1L1 requires further investigation in the future.

## Conclusion

Curcumin, the dietary polyphenol isolated from turmeric can inhibit cholesterol uptake in the enterocytes by inhibiting the expression of NPC1L1, the key transporter of cholesterol in the cell membrane.

## Competing interests

The authors declare that they have no competing interests.

## Authors' contributions

DF is the major investigator involved in the bench work, data acquisition, analysis and manuscript preparation. LO and RDD are involved in the design and organization of the study, interpretation of the results, and the preparation of the manuscript. All authors have read and approved the final manuscript.
